# Productivity or discrimination? An economic analysis of excess-weight penalty in the Swedish labor market

**DOI:** 10.1007/s10198-014-0611-7

**Published:** 2014-06-06

**Authors:** Margareta Dackehag, Ulf-G. Gerdtham, Martin Nordin

**Affiliations:** 1 Department of Economics, Lund University, P.O. Box 7082, 220 07 Lund, Sweden; 2 Health Economics and Management, Institute of Economic Research, Lund University, P.O. Box 7080, 220 07 Lund, Sweden; 3 Centre for Primary Health Care Research, Lund University, Lund, Sweden

**Keywords:** Sweden, Income, Obesity, Overweight, Productivity, Discrimination, I10, I18, J23, J31

## Abstract

This article investigates the excess-weight penalty in income for men and women in the Swedish labor market, using longitudinal data. It compares two identification strategies, OLS and individual fixed effects, and distinguishes between two main sources of excess-weight penalties, lower productivity because of bad health and discrimination. For men, the analysis finds a significant obesity penalty related to discrimination when applying individual fixed effects. We do not find any significant excess-weight penalty for women.

## Introduction

Obesity rates in Western countries over the past 30 years have increased rapidly [1], and Sweden is not an exception: In Sweden, the share of overweight and obese among men aged 16–84 years has increased from 30 % to more than 50 %; for women, the share has increased from 25 to 35 % (Statistics Sweden, Survey of Living Conditions). The general picture emerging from research on excess weight and labor market outcomes states that heavy individuals, particularly women, are less likely to participate in employment and tend to earn less [2–22]. However, the results are not conclusive. For example, Norton and Han [23] do not find any negative weight effect on labor market outcomes for American men and women. Similarly, Behrman and Rosenzweig [24] do not observe any weight penalty in wages for US women. Another exception is presented by Brunello and D’Hombres [25], who observe that the negative effect on wages is stronger for men than for women, using data from the European Community Household Panel. Cawley [9] argues that the mixed results are partly a consequence of different identification strategies. The type of weight measure is another factor that could influence results [7].

The literature discusses two main channels through which excess weight may influence labor market outcomes: lower productivity due to bad health and discrimination. Obesity and overweight are associated with comorbidities such as type II diabetes, various types of cancer and cardiovascular diseases [26, 27], conditions that may contribute to reducing individual ability to work [16, 28–34]. Furthermore, an association between lower productivity and excess weight on average may pose difficulties for the individual of excess weight to get hired or get a pay raise. Apart from such statistical discrimination, excess weight individuals are also the targets of discriminatory attitudes that ascribe negative characteristics, e.g., laziness and lack of self-discipline, to them [35, 36]. Such inferences, based on physical attractiveness, appear to carry over to the labor market [18, 37–39].

Although the channels are often difficult to separate, it is important for studies investigating discrimination to account for potential productivity losses due to bad health.^1^ Studies that investigate the impact of health when analyzing the relationship between excess weight and labor market outcomes observe different effects. Baum and Ford [10], using US data, Morris [4], using UK data, and Greve [3], analyzing Danish data, conclude that health measures have limited influence on the excess weight penalties found in their analyses. By contrast, Lundborg et al. [40] observe a very strong effect of health on the income penalty for excess weight among Swedish men. The mixed findings motivate further research on the influence of health on excess weight penalties in the labor market.

This article investigates the excess-weight penalty in income for men and women in the Swedish labor market, using longitudinal data. Previous studies using Swedish data are rare, and, to our knowledge, we provide the first analysis of weight and income for Swedish women. We regress income on lagged weight categories, applying different identification strategies, OLS and individual fixed effects. The former strategy considers the impact of weight, e.g., the impact of being obese, while the latter considers the impact of changes in weight, e.g., the impact of becoming obese. We distinguish between the productivity and discrimination channels by controlling for individual health, using several measures. Any remaining excess-weight penalty is considered an indication of potential discrimination. In addition, our approach allows us to explore the “health effect” across identification strategies.

The article is outlined as follows. The next section discusses the data and variables and presents descriptive statistics of the sample. The “Methods” section contains a discussion of methodological issues, while the “Results” section presents the estimates from our main analysis as well as sensitivity analyses. The “Discussion” section discusses the results, and the “Conclusion” section concludes the article.

## Data and descriptive statistics

Our empirical analysis uses data from the Swedish Survey of Living Conditions (the ULF survey). The ULF survey is an annual systematic survey of living conditions conducted by Statistics Sweden since 1975. The data are collected during 1-h personal interviews with randomly selected individuals aged 16–84 years and complemented with information from various registers. On average 7,500 individuals are interviewed yearly. The database is primarily cross-sectional, but it also contains a longitudinal panel. The panel is complemented with immigrants and young individuals who have become old enough to be included in the population [41]. The questions are divided into four main themes: Health, Social relations, Physical environment and Work. The survey always contains some central questions from all themes. However, every 8 years each theme receives particular attention. This study uses unbalanced panel data from four 2-year waves, 1980–1981, 1988–1989, 1996–1997 and 2004–2005, covering a 25-year period and focusing on health-related issues. The last two survey waves had 75 % response rates [41].

At the outset, the sample consists of *n* = 22,855 observations. The sample is restricted to working-age individuals, i.e., those aged 20–64 years (*n* = 16,816) who have not retired (*n* = 15,779). We are only interested in individuals who appear at least twice (*n* = 10,048). The lag length in the final sample varies between 8 years and 16 years (<5 % of the sample observations). In addition, we require information on BMI and that BMI is lower than 45, thereby including individuals who are morbidly obese (12 observations) but excluding individuals who are super obese (3 observations).^2^ Those who are or have been underweight are also excluded (*n* = 9,591), making normal weight the reference group for the two excess-weight categories, overweight and obese.^3^ Furthermore, we eliminate missing observations regarding education (*n* = 9,570) and health measures (*n* = 9,567). The final requirement states that individuals must be employed and have a relatively strong connection to the labor market (thereby avoiding the analysis of individuals who work very little during a year, e.g., those who only have a summer job). We code this requirement as annual income from employment exceeding at least 100,000 SEK (approximately $15,750). Our final sample consists of *n* = 8,214 observations belonging to 2,415 men and 2,184 women (*N* = 4,599).

### Dependent variables

This article examines the association between excess weight and income, measured as the logarithm of annual income from employment, above a threshold of 100,000 SEK. Income from employment is based on tax records and includes salaries and benefits such as sickness, unemployment and parental leave benefits. Benefit payments are conditioned on labor market activity, and the amount is related to the individual income level. If benefit payments mask differences in behavior related to weight, our analysis will generate biased results. This concern is particularly related to women, who tend to allocate more time to the care of children and of the home, and also tend to suffer from worse health than men, factors that may all affect labor supply negatively (see, e.g., [44–46]). Unfortunately, there is no measure of income from employment that excludes benefit payments available to us.^4^ The consequence for our analysis is most likely an overestimation of the obesity penalty for women. However, in lieu of an income measure excluding benefits, we specify several health variables that should pick up conditions and circumstances that could influence both weight and income. We also include a control for having small children when analyzing excess-weight penalties for women, thereby taking account of any differences in the family situation that could influence the income level of excess-weight women.^5^ Another factor that may influence the results for women is the income threshold itself. If women have a weaker connection to the labor market, it is possible that the excess weight penalty for women is not observable above the income threshold. We investigate this possibility in the “Sensitivity analyses” section where we perform various sensitivity analyses.

Our income measure is the product of the wage rate and the number of hours worked during a year. In consequence, any indication of income penalties due to excess weight may be associated with either fewer work hours or a lower wage rate or both. However, Antelius and Björklund [47], studying the returns on education in Sweden, observe that the analysis when excluding annual income below 100,000 SEK generates results that are similar to those obtained in an analysis of hourly income. To the degree that this relationship holds in other contexts, our analysis will contribute to elucidating the association between excess weight and wage rates for Swedish employees (see also Lundborg et al. [40], who apply the same income threshold when analyzing obesity and income for Swedish men). In addition, we have run regressions controlling for hours of work per week without observing any marked differences in our main results.

### Independent variables

#### Excess-weight measures

We measure normal weight, overweight, and obesity using BMI, based on self-reported weight and height. This article relies on the WHO classification of weight categories: normal weight is a BMI of 18.5–25, overweight 25–30 and obesity ≥30.

#### Additional background variables

We control for individual age, age squared, and whether or not the individual is married or cohabiting, respectively. We also control for first generation immigrant status or second-generation immigrant status [born in Sweden by parents, one of which is or both are non-Swedish citizen(s)]. Pregnancy tends to increase weight and decrease income (due to work reduction during pregnancy and after birth). These pregnancy-related effects could bias the estimates for women, implying an amplification of the excess-weight penalties. Unfortunately, we cannot exclude pregnant women from the analysis because the ULF survey does not collect information about pregnancy at the time of the interview. However, the survey collects information about how many children the respondent has in different age ranges (0–6 years, 7–18 years, 0–12 years, etc.). Thus, in lieu of information about pregnant respondents, we use a dummy variable describing whether or not the individual has small children, aged 6 years or younger. (We also try using lagged values of the children dummy in the analysis without observing any material changes in the weight estimates.) The analysis also considers four levels of educational attainment, in the form of dummy variables: primary school, 2 years of secondary school, more than 2 years of secondary school and higher (post-secondary) education. In addition, we control for panel waves and region of residence; living in northern or southern Sweden, or in a large city (Stockholm, Gothenburg or Malmoe).

We use a set of variables to control for health: (1) self-assessed health, (2) pain or discomfort due to disease(s), (3) anxiety, nervousness and uneasiness, and (4) mobility. Self-reported health functions as the general measure of health, while the other measures reflect different dimensions of health; the impact of suffering from a disease, of mental health status and of physical ability. In the first two waves, the measure of self-assessed health uses a three-point scale (“good,” “between good and bad” and “bad”). In the last two waves, the measure uses a five-point scale (“very good,” “good,” “between good and bad,” “bad” and “very bad”). We construct a measure of self-assessed health using the three-point scale, merging assessments of “very good” and “very bad” health into the categories of “good” and “bad” health, respectively. Bad health receives the lowest score (1) and good health the highest score (3).^6^ In the ULF survey, respondents are asked to specify up to six diagnoses from which they suffer and to assess the pain or discomfort experienced because of each diagnosis. Based on the reported frequency and intensity of the pain or discomfort, we construct a measure that ranks the pain along a three-point scale, where high levels of pain receive the highest score (3) and low levels of pain receive the lowest score (1). The variable measuring anxiety, nervousness and uneasiness is also constructed in the same way: a three-point scale indicating severe problems by the highest score (3) and no problems by the lowest score (1). The mobility variable indicates whether the respondent can run a short distance when necessary (e.g., when trying to catch a bus). Table 1 provides the descriptive statistics of our sample.

**Table 1:** Descriptive statistics

	Men		Women	
	Mean	SD	Mean	SD
Number of observations	4,349		3,865	
Number of individuals	2,415		2,184	
Annual labor market income (in hundreds of SEK)	2,832.30	1,495.48	2,050.99	803.69
Log annual labor market income	7.87	0.38	7.57	0.33
Normal weight (reference)	0.51	0.50	0.66	0.47
Overweight	0.42	0.49	0.27	0.44
Obese	0.08	0.27	0.07	0.25
Primary education (reference)	0.19	0.39	0.15	0.36
1–2 years of secondary education	0.33	0.47	0.36	0.48
>2 years of secondary education	0.16	0.37	0.11	0.32
Higher education	0.31	0.46	0.37	0.48
Alone (reference)	0.25	0.43	0.23	0.42
Married	0.53	0.50	0.56	0.50
Cohabitation	0.22	0.42	0.22	0.41
Age	42.78	10.66	43.11	10.70
Small children			0.21	0.41
Health	2.83	0.43	2.80	0.46
Pain	1.31	0.57	1.36	0.61
Anxiety	1.10	0.34	1.17	0.43
Mobile	0.96	0.19	0.94	0.24
Non-immigrant (reference)	0.88	0.33	0.86	0.34
1st generation immigrant	0.06	0.24	0.08	0.27
2nd generation immigrant (2)	0.01	0.11	0.02	0.12
2nd generation immigrant (1)	0.05	0.22	0.05	0.21
Northern Sweden (reference)	0.19	0.39	0.19	0.39
Southern Sweden	0.50	0.50	0.50	0.50
Large city	0.31	0.46	0.31	0.46
Wave 1988–1989 (reference)	0.35	0.48	0.32	0.47
Wave 1996–1997	0.35	0.48	0.35	0.48
Wave 2004–2005	0.30	0.46	0.33	0.47

Means calculated for waves 1988–1989, 1996–1997 and 2004–2005

Attrition bias is a potential problem because individuals with certain characteristics may drop out of the panel between the survey waves. We investigate the extent of the attrition bias by comparing the variable means in the panel sample, separated into three groups. Group 1 contains observations belonging to individuals appearing once in the sample and group 2 contains observations belonging to individuals appearing twice in the sample. Because of our use of lagged weight variables, single and double appearances imply that the individuals have responded twice and three times respectively in the survey. In these two groups there are individuals who have responded on all possible occasions, individuals who have not responded on one or two occasions as well as individuals whose responses are excluded from our sample because of the age restrictions we set up. Group 3 contains the observations of individuals appearing three times in the sample, i.e., responding in all four survey waves. Table 4 in Appendix 1 shows the variable means per group (the first three columns) and presents the *p* values of the *t* tests when we compare the groups pairwise: group 1 to group 2, group 2 to group 3 and group 1 to group 3 (the last three columns). Generally, when we compare the *p* values of the pairwise *t* tests, we observe that the characteristics of group 1 differ significantly at 5 % from the other two groups (column 4 and 6) more often than the characteristics of group 2 compared to group 3 (column 5). Focusing on the comparison of group 1 and group 3 (last column), we note that among other things group 1 tends to earn less, have invested less in higher education (significant at 10 %), have worse health, be less overweight, be younger and have more immigrant representation. Notably, there is no significant difference in average obesity. In fact, across all three groups we observe increasing average income, as well as age, but no significant differences in obesity. However, group 3 is significantly more overweight on average. Partly these observations may indicate a positive relationship among age, income and weight. Indeed, when studying the means for income and weight variables of group 3, while decreasing the maximum age limit, we find that the means become more like the ones of group 1. Overall, we find little indication that attrition bias is a major problem for our analysis.^7^

## Methods

In similarity with Cawley [9] and other studies [8, 11–13, 17, 23, 40], we use lagged BMI (classified as normal weight, overweight and obese) as a means to control for reversed causality.

The use of two identification strategies allows us to consider different aspects of the relationship between weight and income. While the OLS approach analyzes the impact on current income of lagged weight, the fixed effects approach analyzes the impact on current income of changes in lagged weight.

We estimate the weight impact on income by applying OLS on a pooled data model. Equation 1 shows the baseline model:

1 $$\ln \left( {y_{it} } \right) \, = \, \beta W_{it - 1} + \, \gamma X_{it} + \, \delta T_{t} + \, \varepsilon_{it}$$

where *y* is annual income, *W* is a vector of dummy variables indicating overweight and obesity, and *X* is a vector of explanatory variables, including marital status, education attainment, etc., for individual *i* at time *t*. The vector *T* contains panel wave dummies. We assume that the error term *ε* is random and uncorrelated with the explanatory variables, zero mean and constant variance. To control for health-related productivity reduction, we analyze a second model:

2 $$\ln \left( {y_{it} } \right) \, = \, \beta W_{it - 1} + \, \gamma X_{it} + \theta H_{it} + \, \delta T_{t} + \, \varepsilon_{it}$$

where *H* is a vector of health variables.^8^ If the analysis produces significant excess weight estimates, we attribute the remaining excess weight penalty to discrimination (cf., [3, 10, 40]). However, to the extent that other unobservable characteristics, e.g., self-confidence or time preferences [10, 15], influence the relationship between weight and income, the OLS approach generates biased results. In other words, there is a risk that we overestimate the impact of discrimination as a channel through which excess weight is penalized in the labor market.

Taking advantage of the panel, we can control for individual time-invariant unobserved heterogeneity, which may otherwise bias the OLS estimates.^9^ Among studies investigating the excess weight penalty in the labor market by using (individual or sibling/family) fixed effects [3, 9–12, 24], Lundborg et al. [40] represents another example where current earnings are regressed on lagged BMI. The baseline model, using individual fixed effects, is:

3 $$\ln \left( {y_{it} } \right) \, = \, \beta W_{it - 1} + \, \gamma X_{it} + \, \mu_{i} + \, \lambda_{t} + \, \varepsilon_{it}$$

where *y*, *X* and *ε* are defined as in Eq. 1, *μ* is the individual-level fixed effect, and λ the time fixed effect, which we estimate using panel wave dummies. Assuming that explanatory variables in vector *X* and *μ* are correlated, the fixed effects approach uses the difference within individual observations over time to eliminate μ. We add health-related variables in the second fixed effects model, as shown by Eq. 4:

4 $$\ln \left( {y_{it} } \right) \, = \, \beta W_{it - 1} + \, \gamma X_{it} + \, \theta H_{it} + \, \mu_{i} + \, \lambda_{t} + \, \varepsilon_{it.}$$

Our analysis is based on self-reported BMI, which may be subject to measurement error, as (excess weight) respondents tend to under-report weight and over-report height. Under-reporting of BMI also depends on gender and age; women and younger individuals are found to underreport BMI more than men and older individuals [48–51]. Socioeconomic status is another factor that may influence misreporting [52, 53]. Some studies correct for misreporting by using fitted values based on anthropometric data [9, 54–56]. Another method to deal with reporting error involves lowering the threshold for obesity [49, 57, 58]. Having no access to anthropometric data, we adopt the latter method and investigate the effect of changing the cutoff point by gender in “Sensitivity analyses” section.

Excess weight penalties may work through sorting in different dimensions and stages of life, e.g., education, occupation and on the marriage market [15, 40, 59, 60]. Among the explanatory variables we include educational choices and family status but exclude variables relating to work. In other words, our aim is to analyze the impact of excess weight in the labor market specifically, not the total effect of excess weight.

In the fixed effects approach, we cannot control for (linear) age and time simultaneously, because age is a function of time, *β*age_*it*_ = *β*age_*i*0_ + *βt* [61]. The first RHS term is time-invariant and will disappear when we apply individual fixed effects. The second RHS term is identical for all individuals at time *t* and will be picked up by the panel wave dummies. We drop linear age from the model specifications but keep age squared in the fixed effects framework. Thus, the panel wave dummies reflect the cohort effects, while age squared captures the income effect associated with increasing age. We do not observe any considerable changes in the estimates when excluding age squared and keeping the time dummies.

## Results

### Income

Table 2 summarizes the results from the income regressions for both genders. Appendix 2 contains tables (Tables 5, 6) showing the estimates for the full model specifications. We use two identification strategies, OLS and individual fixed effects, and present the estimation results in that order. For each strategy there are two model specifications, a baseline model (column 1 for OLS and column 3 for fixed effects, FE) that contains individual background variables including educational attainment and a second model (column 2 for OLS and column 4 for FE) that adds health variables. Starting with the OLS results for men (columns 1 and 2 in Table 2), we observe in the baseline a 6 % obesity penalty (*p* < 0.10). When we take differences in health into account, the penalty decreases to <4 % and loses statistical significance. By contrast, overweight men do not appear to experience lower annual income compared to their normal-weight peers. The penalty is very small and statistically insignificant in the baseline and disappears in the second model containing health variables. For obese women, the baseline OLS estimate reveals a statistically insignificant penalty amounting to 1.3 %, a penalty that is erased in the second model. We find no indication of income differences due to overweight for women.

**Table 2:** Income and excess weight

	OLS		FE	
	(1)	(2)	(3)	(4)
Men				
Obese	−0.0596* (0.0331)	−0.0374 (0.0315)	−0.0956*** (0.0344)	−0.0916*** (0.0347)
Overweight	−0.00318 (0.0130)	0.00154 (0.0128)	−0.00972 (0.0155)	−0.00828 (0.0155)
*R* ^2^	0.261	0.282	0.349	0.353
Women				
Obese	−0.0126 (0.0246)	0.00458 (0.0249)	−0.0238 (0.0424)	−0.0173 (0.0403)
Overweight	−0.00343 (0.0122)	0.00353 (0.0121)	0.000395 (0.0165)	0.00112 (0.0166)
*R* ^2^	0.306	0.321	0.453	0.461

Men (*n* = 4,349, *N* = 2,415) and women (*n* = 3,865, *N* = 2,184)

Robust standard errors in parentheses, *** *p* < 0.01; ** *p* < 0.05; * *p* < 0.1. Estimation with OLS and individual fixed effects

The dependent variable measures the logarithm of annual labor market income exceeding a minimum of 100,000 SEK annually. Model 1 (first and third columns) controls for lags of obesity and overweight (using normal weight as reference), age (only in OLS) and age squared, marital status, cohabitation, being a first- or second-generation immigrant, education, region of residence and panel wave. For women, the baseline model also contains a variable saying whether or not the individual has children aged 6 years or younger. Model 2 (second and fourth columns) adds controls for self-assessed health, pain or discomfort due to disease, anxiety and mobility

Columns 3 and 4 in Table 2 show the fixed effects estimates. The results for obese men are quite strong. In the baseline model, we observe an obesity penalty of 9.6 % (*p* < 0.01). The penalty proves to be quite robust to controls for health; it decreases to 9.2 % and remains strongly significant. By contrast, the overweight penalty for men is roughly 1 % and insignificant in both models. We do not observe any significant excess weight penalties for women. The obesity penalty amounts to 2.4 % in baseline and falls below 2 % in the second model. The overweight estimates for women are positive insignificant in both models. Overall, the influence of health on the weight penalty appears to be smaller in the fixed effects framework than in the OLS framework.^10^

### Sensitivity analyses

#### Adjustment of the obesity threshold

We investigate whether our results are affected by survey respondents misreporting their height and weight. We apply lower, gender-specific obesity thresholds: BMI ≥29.5 for men and BMI ≥29 for women. The adjustments are based on the findings of Boström and Diderichsen [62], who analyze the misclassification of BMI using questionnaire data on individuals living in Stockholm county, Sweden. They observe an underestimation of BMI that differs by gender: −0.85 for women and −0.4 for men.

Table 7 in Appendix 3 presents the excess weight estimates from the income regressions with the new obesity thresholds. For men, the lower obesity threshold changes the magnitude of the weight estimates in different directions depending on the identification strategy but does not materially alter the results from our main analysis. Thus, we find no clear support for BMI measurement error for obese men. For women, the threshold adjustment implies small changes regarding size in the OLS results, which remain insignificant. When inspecting the fixed effects estimates, we observe a large increase in magnitude but no change in statistical significance. The indications of a BMI misclassification are inconclusive also in this case.

#### Changes in the income threshold

We investigate the effect of removing the requirement of a minimum annual income of 100,000 SEK, thereby shifting focus from income in terms of hourly wages to income in terms of hours worked (see [47]). Thus, we are able to say something about the excess-weight penalty among individuals with a weak connection to the labor market. Table 3 summarizes the regression results. The results indicate a stronger influence of excess weight on income, in particular for obese men. In the OLS baseline, the obesity penalty for men amounts to 15.5 % (*p* < 0.05). When we control for health, the penalty decreases to roughly 13 % (*p* < 0.10). There is (still) no association between overweight and lower income among men in the OLS framework. Continuing with the fixed effects results for men, we observe a baseline obesity penalty of 16.6 % (*p* < 0.05). The second model produces a penalty of almost the same magnitude, 16.5 %, and of the same statistical significance. In addition, we observe an overweight penalty of almost 2 %, but the difference in relation to their normal-weight peers is insignificant in both models. When analyzing weight and income for women, we find that the OLS estimates are larger compared to those in our main analysis (see Table 2) but remain insignificant. The baseline estimates imply that excess weight decreases income by more than 4 % on average. Furthermore, the overweight penalty is larger than the obesity penalty, a relationship that holds in the second model. Concerning the fixed effects approach, the inclusion of low-income earners in the analysis alters neither estimate size nor significance level; we find no indication of excess-weight penalties for women.^11^

**Table 3:** **Income and excess weight, no income threshold**

	OLS		FE	
	(1)	(2)	(3)	(4)
Men				
Obese	−0.155** (0.0752)	−0.136* (0.0767)	−0.166** (0.0714)	−0.165** (0.0725)
Overweight	0.00318 (0.0249)	0.00849 (0.0246)	−0.0178 (0.0377)	−0.0175 (0.0379)
*R* ^2^	0.078	0.088	0.121	0.122
Women				
Obese	−0.0405 (0.0570)	−0.0198 (0.0572)	−0.0294 (0.0790)	−0.0183 (0.0804)
Overweight	−0.0458 (0.0300)	−0.0344 (0.0297)	0.00496 (0.0353)	0.00605 (0.0353)
*R* ^2^	0.103	0.113	0.176	0.179

Men (*n* = 4,686, *N* = 2,572) and women (*n* = 4,449, *N* = 2,418)

Robust standard errors in parentheses, *** *p* < 0.01; ** *p* < 0.05; * *p* < 0.1. Estimation with OLS and individual fixed effects. The dependent variable measures the logarithm of annual income from employment >0 SEK. For full model specification, see Table 2

Including annual income below 100,000 SEK in the analysis, we observe a strong obesity penalty for men. The result implies that obese men with low income work less than their normal-weight peers. However, we cannot rule out that the penalty is (partly) mediated through the wage rate (cf., [40]). The analysis also shows that the obesity penalty is relatively insensitive to controls for health using both identification strategies, a relationship that indicates that health-related productivity reductions do not drive the results (although the statistical significance is weak in the OLS approach; see Table 3, column 2).

## Discussion

When analyzing the impact of excess weight on income from employment for Swedish men and women, we observe that men appear to experience an obesity penalty while women do not. When we regress income (of at least 100,000 SEK annually) on weight using OLS, the obesity penalty for men reaches almost 6 %. Using individual fixed effects, we find that the penalty amounts to more than 9 %. Considering the larger penalty found when applying the second identification strategy, individual fixed effects, we conclude that (factors inducing) changes in weight (categories) have a particularly strong influence on income for men, while permanent excess weight (considered in the OLS analysis) does not. However, for men with a weak connection to the labor market differences in “weight profiles” have little influence on the obesity estimates. Analyzing income and weight without the income threshold, we find that obese men experience on average a 14–16 % penalty in annual income irrespective of identification strategy. Our results contrast with previous studies that apply both OLS and individual fixed effects; they find that taking individual fixed effects produce smaller weight estimates compared to OLS [9, 10]. However, these studies do not use lagged weight when applying fixed effects, implying that there may be a problem of reversed causality diluting the negative effect of weight on income. Indeed, when we regress current income on current weight applying individual fixed effects, we observe large, positive, but insignificant obesity estimates (results not shown).

Compared to most other studies finding significant obesity penalties for men, our estimates are large. Baum and Ford [10] observe a significant obesity penalty of <1 % for men in the US, using individual fixed effects and current weight as independent variable. Our results also contrast with the IV estimates in Brunello and D’Hombres [25], who find that obese men earn significantly (3.3 %) less on average, using data from nine European countries (Sweden not included). However, our findings are fairly in line with another analysis of Swedish data. Lundborg et al. [40], who investigate the relationship between current income and excess weight at the age of 18 for Swedish men aged 28–38 years, find considerable and significant effects of excess weight, approximately 9 % in the baseline, using sibling fixed effects. Together these studies indicate that the obesity penalty for men may be relatively large in the Swedish labor market. However, further research on the Swedish labor market is needed to corroborate our results.

We also find that health measures appear to impact differently on the obesity penalty depending on the identification strategy. The OLS results indicate that obese men have worse health and therefore earn less. When we account for individual fixed effects, health is less influential. This result implies that lower productivity is not the main suspect when searching for the source of the obesity penalty. Instead, discrimination may be an important underlying determinant. The difference in impact may again reflect the two identification strategies picking up different properties of the sample population; (bad) health may be an influential factor in explaining the labor market outcome for an individual who already *is* obese, but when it comes to income and *changing* weight categories, health may not be a main driver. We find that the relationship between “health effect” and identification strategy is less pronounced when including low-income earners in the analysis (see Table 3), a finding that may indicate that discrimination is a bigger problem for individuals with a weak connection to the labor market, irrespective of constant or changing weight. In similarity with most studies investigating the impact of health on excess-weight penalties, we find a limited influence of health. Lundborg et al. [40] come to a different conclusion. However, they rely on anthropometric data instead of self-reported data when constructing their health measures (e.g., cardiovascular fitness), which may explain the different results.

Contrary to many previous studies, we find that excess weight is not a problem for women concerning income. However, our dependent variable, income from employment, includes income-related benefits, e.g., sickness benefits, which may conceal ways in which weight may influence income. In lack of another income measure, we try to mitigate the problem of measurement error by including controls for several health measures and having small children (as well as labor market status, see footnote 5). As recent research indicates, it is possible that the barriers for heavy women rather exist at the employment stage in the Swedish labor market [38]. Moreover, small and insignificant estimates for women may be an effect of measurement error due to survey respondents underreporting their BMI. We try to correct for that possibility by adjusting the obesity threshold downwards. However, in similarity with other studies adopting that method, our results remain, on the whole, the same [49, 57, 58]. Mitigating the potential problem of BMI measurement error, recent research implies that misreporting may be a decreasing problem because of changes in social norms related to weight [63]. Self-reported or not, BMI may be a flawed measure of obesity, since it does not distinguish between fat mass and fat-free mass. Recent studies on obesity and labor market outcomes use alternative indicators based on body composition, e.g., fat mass and waist circumference [7, 20, 64, 65]. It is possible that our weight estimates for women would alter if we had the opportunity to use such measures. For example, Johansson et al. [7] observe a negative association for Finnish women between income and waist circumference, but not between income and BMI.

## Conclusions

This study investigates the relationship between excess weight and income for men and women in the Swedish labor market. Our analysis shows that there is a significant obesity penalty for men, but not for women; a reverse gender pattern relative to the one found in the majority of studies analyzing excess weight and labor market outcomes. In addition, we find that the obesity penalty for men is considerable in magnitude and relates to discrimination rather than lower productivity due to bad health. Our findings fit with previous research of the Swedish labor market, but considering that there are only a few studies to date, the picture of how weight influences labor market outcomes in Sweden is incomplete. Further research on weight, labor market outcomes and gender in the Swedish labor market is clearly warranted.

## Appendix 1

See Table 4

**Table 4:** Sample attrition analysis by the number of appearances in the full sample

	Group 1	Group 2	Group 3	*t* test Gr 1 versus Gr 2	*t* test Gr 2 versus Gr 3	*t* test Gr 1 versus Gr 3
	Mean	Mean	Mean	*p* value for H0 of equal means	*p* value for H0 of equal means	*p* value for H0 of equal means
Labor market income (in hundreds of SEK)	2,312.81	2,437.28	2,592.86	0.00	0.00	0.00
Obese	0.03	0.04	0.03	0.88	0.86	0.99
Overweight	0.22	0.23	0.26	0.57	0.01	0.01
1–2 years of secondary school	0.29	0.36	0.37	0.00	0.50	0.00
>2 years of secondary school	0.18	0.14	0.12	0.00	0.05	0.00
Higher education	0.33	0.33	0.36	0.66	0.02	0.09
Health	2.77	2.82	2.85	0.00	0.00	0.00
Pain	1.38	1.34	1.30	0.05	0.00	0.00
Mobile	0.93	0.95	0.96	0.02	0.12	0.00
Anxiety	1.17	1.14	1.10	0.02	0.00	0.00
Married	0.49	0.55	0.57	0.00	0.05	0.00
Cohabiting	0.25	0.22	0.20	0.01	0.16	0.00
1st generation immigrant	0.10	0.06	0.05	0.00	0.33	0.00
2nd generation immigrant (2)	0.01	0.01	0.02	0.36	0.13	0.68
2nd generation immigrant (1)	0.05	0.05	0.05	0.28	0.80	0.20
Age	41.80	42.84	43.77	0.00	0.00	0.00
Male	0.51	0.54	0.53	0.04	0.48	0.15
Living in southern Sweden	0.47	0.51	0.52	0.01	0.31	0.00
Living in a large city	0.36	0.30	0.29	0.00	0.52	0.00
Observations *n*	1,999	3,170	3,045			
Individuals *n*	1,999	1,585	1,015			

Group 1 (2, 3) consists of observations belonging to individuals appearing once (twice, three times) in the sample. Means for group-wise samples and *p* values for the null hypothesis of equal means

## Appendix 2

See Tables 5 and 6.

**Table 5:** Full regression results, income and excess weight

	OLS		FE	
	(1)	(2)	(3)	(4)
Obese	−0.0596* (0.0331)	−0.0374 (0.0315)	−0.0956*** (0.0344)	−0.0916*** (0.0347)
Overweight	−0.00318 (0.0130)	0.00154 (0.0128)	−0.00972 (0.0155)	−0.00828 (0.0155)
Health		0.0584*** (0.0131)		0.0280** (0.0128)
Pain		−0.0502*** (0.00935)		−0.0148 (0.0104)
Anxiety		−0.0519*** (0.0151)		0.00231 (0.0159)
Mobile		0.0158 (0.0268)		0.0245 (0.0274)
Married	0.146*** (0.0138)	0.136*** (0.0136)	0.0348* (0.0188)	0.0369** (0.0188)
Cohabiting	0.0749*** (0.0137)	0.0705*** (0.0136)	0.0390** (0.0163)	0.0407** (0.0165)
1–2 years of secondary school	0.0548*** (0.0151)	0.0561*** (0.0147)	0.0172 (0.0182)	0.0221 (0.0185)
>2 years of secondary school	0.137*** (0.0196)	0.137*** (0.0192)	0.0477* (0.0254)	0.0519** (0.0255)
Higher education	0.300*** (0.0194)	0.294*** (0.0190)	0.0648* (0.0345)	0.0678* (0.0349)
1st generation immigrant	−0.141*** (0.0258)	−0.125*** (0.0252)		
2nd generation immigrant (2)	0.0715 (0.0810)	0.0720 (0.0802)		
2nd generation immigrant (1)	−0.0275 (0.0246)	−0.0278 (0.0239)		
Age	0.0402*** (0.00397)	0.0417*** (0.00394)		
Age^2^	−0.000403*** (4.63e−05)	−0.000412*** (4.60e−05)	−0.000556*** (4.50e−05)	−0.000547*** (4.53e−05)
Living in southern Sweden	0.0246 (0.0155)	0.0211 (0.0153)	0.156** (0.0733)	0.151** (0.0722)
Living in large city	0.118*** (0.0186)	0.112*** (0.0184)	0.204** (0.0813)	0.201** (0.0805)
Wave 1996–1997	0.0126 (0.00944)	0.0137 (0.00937)	0.442*** (0.0336)	0.439*** (0.0337)
Wave 2004–2005	0.157*** (0.0120)	0.159*** (0.0118)	1.049*** (0.0666)	1.042*** (0.0668)
Constant	6.614*** (0.0831)	6.518*** (0.0954)	8.281*** (0.0863)	8.179*** (0.104)
*R* ^2^	0.261	0.282	0.349	0.353

Men (*n* = 4,349, *N* = 2,415)

**Table 6:** Full regression results, income and excess weight

	OLS		FE	
	(1)	(2)	(3)	(4)
Obese	−0.0126 (0.0246)	0.00458 (0.0249)	−0.0238 (0.0424)	−0.0173 (0.0403)
Overweight	−0.00343 (0.0122)	0.00353 (0.0121)	0.000395 (0.0165)	0.00112 (0.0166)
Health		0.0366*** (0.0120)		0.0380*** (0.0147)
Pain		−0.0147* (0.00894)		−0.0199* (0.0108)
Anxiety		−0.0574*** (0.0116)		−7.32e−05 (0.0148)
Mobile		0.0260 (0.0205)		0.0473** (0.0234)
Married	−0.0431*** (0.0131)	−0.0516*** (0.0129)	−0.0571*** (0.0215)	−0.0533** (0.0214)
Cohabiting	−0.0301** (0.0148)	−0.0384*** (0.0146)	−0.0399** (0.0195)	−0.0385** (0.0194)
Small children	−0.121*** (0.0129)	−0.124*** (0.0128)	−0.124*** (0.0149)	−0.127*** (0.0148)
1–2 years of secondary school	0.0523*** (0.0136)	0.0468*** (0.0135)	0.00537 (0.0213)	0.00217 (0.0210)
>2 years of secondary school	0.135*** (0.0210)	0.131*** (0.0207)	0.0267 (0.0319)	0.0282 (0.0318)
Higher education	0.268*** (0.0158)	0.257*** (0.0157)	0.0691** (0.0349)	0.0623* (0.0351)
1st generation immigrant	−0.0147 (0.0206)	−0.00587 (0.0201)		
2nd generation immigrant (2)	−0.0198 (0.0413)	−0.0146 (0.0420)		
2nd generation immigrant (1)	0.0549** (0.0254)	0.0638*** (0.0246)		
Age	0.0301*** (0.00348)	0.0307*** (0.00346)		
Age^2^	−0.000298*** (3.93e−05)	−0.000301*** (3.91e−05)	−0.000253*** (4.63e−05)	−0.000247*** (4.58e−05)
Living in southern Sweden	−0.00989 (0.0127)	−0.00875 (0.0126)	−0.0885* (0.0514)	−0.0790 (0.0513)
Living in large city	0.0861*** (0.0156)	0.0866*** (0.0155)	0.0291 (0.0526)	0.0346 (0.0521)
Wave 1996–1997	0.0452*** (0.00862)	0.0471*** (0.00867)	0.255*** (0.0338)	0.255*** (0.0335)
Wave 2004–2005	0.210*** (0.0114)	0.218*** (0.0113)	0.666*** (0.0680)	0.671*** (0.0674)
Constant	6.674*** (0.0754)	6.623*** (0.0899)	7.830*** (0.0775)	7.689*** (0.0989)
*R* ^2^	0.306	0.321	0.349	0.353

Women (*n* = 3,865; *n* = 2,184)

## Appendix 3

See Table 7.

**Table 7:** Income and excess weight, adjusted obesity thresholds

	OLS		FE	
	(1)	(2)	(3)	(4)
Men				
Obese	−0.0681** (0.0291)	−0.0449 (0.0275)	−0.0783*** (0.0298)	−0.0741** (0.0301)
Overweight	0.000212 (0.0130)	0.00411 (0.0129)	−0.0100 (0.0155)	−0.00861 (0.0155)
*R* ^2^	0.262	0.282	0.349	0.353
Women				
Obese	−0.0162 (0.0205)	−0.00179 (0.0206)	−0.0517 (0.0348)	−0.0453 (0.0339)
Overweight	−0.00180 (0.0125)	0.00501 (0.0124)	0.00302 (0.0165)	0.00350 (0.0166)
*R* ^2^	0.306	0.321	0.454	0.462

Men (*n* = 4,349, *N* = 2,415) and women (*n* = 3,865, *N* = 2,184)

Robust standard errors in parentheses, *** *p* < 0.01; ** *p* < 0.05; * *p* < 0.1. Estimation with OLS and individual fixed effects. Gender-specific adjustment of the obesity threshold, BMI ≥29.5 for men and BMI ≥29 for women. For full model specification, see Table 2

## Footnotes

1 There are other underlying factors, e.g., self-confidence and time preferences [e.g., 10, 15] that may influence the relationship between weight and income directly and indirectly through the channels of discrimination and health-related productivity. This study considers the indirect effect when analyzing the excess weight penalty in the Swedish labor market.

2 We exclude super obesity based on a concern for misreported values. Our results are insensitive to the exclusion of these observations.

3 Research on weight and health often finds that underweight is associated with increased health risks, which, however, may differ from those of excess weight [42, 43]. By excluding underweight, our analysis focuses on factors influencing the labor market situation for excess weight individuals. We run regressions including underweight in our model specifications and then observe insignificant underweight estimates and virtually unaltered overweight and obesity estimates.

4 Lundborg et al. [40] find no effect on obesity estimates of excluding social benefits from their income measure, also based on tax records. However, in their case annual income includes earnings from self-employment, which makes it difficult to draw any conclusions pertaining to employed individuals only, considering that there may be a selection problem related to self-employment and excess weight. In addition, their analysis considers men but not women.

5 We also controll for self-reported unemployment and sickness absence, an exercise that does not affect the excess weight estimates in any considerable way.

6 The “health scores” are distributed as follows: about 2.5 % of the observations belong to category “bad health,” about 13 % to “between good and bad health” and about 84.5 % to “good health.” We observe some variation in the distribution over time, e.g., there appears to be a larger share of observations in categories “bad health” and “between good and bad health” (by 1–1.5 % points) in wave 2004–2005 compared to wave 1988–1989. However, whether the variation is attributable to actual changes in health status, to the finer scale introduced in the last wave or to our recoding is difficult to identify.

7 In addition, we also perform an attrition analysis comparing the variable means in a panel sample and a separate cross-section sample for every survey wave (using current values of all variables). Thereby we investigate how representative the panel sample is relative to the cross-sectional one. For the first three waves, there are virtually no significant differences in variable means between the samples. In the fourth wave, we observe several significant differences in variable means (*p* < 0.01). However, there are no significant differences between the two samples with regard to our variables of special interest, income, obesity and health, in any survey wave.

8 There is also a potential concern for reversed causality regarding health and income. However, we have run regressions with lagged weight and lagged health variables without observing any material changes to the excess weight estimates.

9 It is of course possible that time-varying unobservable factors drive the relationship between weight and income. Unfortunately, we do not have any clearly appropriate instrument to perform such an analysis.

10 Investigating the variation of a continuous weight variable (when applying individual fixed effects), we have run regressions of income from employment on BMI and BMI squared in the same model specification. In essence, the analysis of a continuous BMI indicates the same relationship as the analysis of weight categories: We find a positive but decreasing relationship between income and continuous BMI for both men and women, larger estimates for men and significant estimates only for men.

11 We also investigate the effect on the excess weight penalty of applying different income thresholds (annual income >0, >20,000 SEK, >50,000 SEK, >100,000 SEK, >150,000 SEK, >200,000 SEK). Overall, we observe that the obesity penalty decreases in size, in particular the OLS estimates, which also lose considerably in statistical significance when we increase the threshold. We also try applying an income ceiling (maximum 500,000 SEK) and find that the results are quite similar to the ones presented in Table 3. Thus, the relationship between weight and income appears to be robust to the exclusion of very high income (cf., [40]).
